# Predictive Factors for Altered Quality of Life in Patients with Type 2 Diabetes Mellitus

**DOI:** 10.3390/jcm13154389

**Published:** 2024-07-26

**Authors:** Oana Albai, Adina Braha, Bogdan Timar, Romulus Timar

**Affiliations:** 1 Department of Second Internal Medicine—Diabetes, Nutrition, Metabolic Diseases, and Systemic Rheumatology, “Victor Babes” University of Medicine and Pharmacy, 300041 Timisoara, Romania; albai.oana@umft.ro (O.A.); bogdan.timar@umft.ro (B.T.); timar.romulus@umft.ro (R.T.); 2 Department of Diabetes, Nutrition and Metabolic Diseases Clinic, “Pius Brînzeu” Emergency Clinical County University Hospital, 300723 Timisoara, Romania; 3 Centre for Molecular Research in Nephrology and Vascular Disease/MOL-NEPHRO-VASC, “Victor Babes” University of Medicine and Pharmacy, 300041 Timisoara, Romania

**Keywords:** quality of life, type 2 diabetes mellitus, complications, glycemic control

## Abstract

**Objectives**: To evaluate the quality of life (QoL) in a group of patients with type 2 diabetes (T2DM) and to identify predictive factors to apply the necessary measures to improve it. **Methods**: For this, 299 patients with T2DM were enrolled in a cross-sectional study, and their QoL was assessed using the EQ-5D-3L questionnaire. All patients underwent clinical exams, routine laboratory tests, and nerve conduction velocity (NCV) at the common peroneal nerve. **Results**: Patients had a median age of 66 (57; 70) years, median duration of T2DM of 10 (6; 15) years, median HbA1c of 8 (7; 9.3)%, and mean EQ-5D-3L score of 55%. In addition, 9.7% presented extreme difficulty in mobility, 18.5% severe difficulty in self-care, and 16.4% in usual activities. One-third presented with severe pain or discomfort, anxiety, or depression (level 3 EQ-5D-3L). DPN, heart failure (HF), cerebral stroke, and insulin therapy increased the likelihood of a reduced QoL (EQ-5D-3L < 50). The EQ-5D-3L score inversely correlated with serum creatinine, glycemic control, lipid profile, diabetes duration, age, mobility, self-care, pain/discomfort, usual activities, and anxiety/depression and positively correlated with NCV, HDLc, and eGFR. **Conclusions**: Preventing neuropathic complications, chronic kidney disease, stroke, and HF and obtaining the glycemic and lipid targets could improve the QoL in patients with T2DM.

## 1. Introduction

According to the World Health Organization (WHO), quality of life (QoL) refers to individuals’ perceptions of their social situations in the context of the cultural value systems in which they live, depending on their needs, standards, and aspirations. In medicine, QoL means physical, mental, and social well-being and the patient’s ability to perform daily activities [1,2].

The criteria used to assess the QoL differ from person to person and include the following aspects: • Physical Health: general health, the absence of disease or chronic conditions, and the ability to lead an active and healthy lifestyle; • Mental Health: psychological well-being, absence of stress, anxiety, and strength and ability to manage emotional challenges; • Social Relationships: the quality of personal relationships, including family, friends, and community, and the sense of belonging and social support; • Material Well-Being: standard of living, financial security, access to resources, and ability to meet basic needs; • Career and Personal Achievements: satisfaction with career and personal achievements, both professional and personal; • Education and Personal Development: opportunities for learning, development, and personal growth; • Leisure and Recreation: the ability to have time for enjoyable activities and relaxation; • Environment: the quality of the environment in which you live, including the level of pollution, access to green spaces, and the quality of urban or rural life; • Meaning and Purpose: the feeling of having a purpose or direction in life and being able to fulfill your potential; and • Freedom and Autonomy: the degree of personal freedom and autonomy in making decisions and controlling your life [3,4,5].

Diabetes mellitus (DM) is a frequent condition of the endocrine system that appears due to a deficiency of insulin secretion or the resistance of peripheral tissues to its action. DM is a leading cause of death and disability worldwide, affecting people irrespective of country, age group, or sex [6,7]. Genetic susceptibility, an unhealthy diet rich in fats and carbohydrates, vitamin D deficiency, a sedentary lifestyle, weight gain, smoking, excessive alcohol consumption, and stress are among the primary causes of this disease [8,9,10,11,12,13].

DM is a major health challenge of our century due to its increasing prevalence and serious complications. DM complications are acute (hypoglycemia, ketoacidosis, and hyperosmolar hyperglycemic coma) and chronic. DM causes microangiopathic complications: retinopathy leading to blindness, nephropathy with chronic kidney disease in terminal stages, and renal replacement. Also, DM is considered the “equivalent of cardiovascular disease”, increasing the cardiovascular risk two to four times through multiple macroangiopathic complications: stroke, coronary heart disease, cardiomyopathy, heart failure, and peripheral arterial disease [14]. Moreover, liver steatosis is another prevalent comorbidity in about 70% of the patients with type 2 DM (T2DM), which can lead to liver fibrosis and, eventually, cirrhosis [15]. Beyond diabetic retinopathy, diabetic patients with longer diabetes duration could present first with increasing intraocular pressure, with some developing glaucoma in about 8% of cases. However, it is notable to mention that more than 40% of the cases develop cataracts at a younger age than patients without diabetes, increasing even more the risk of blindness [16]. Another frequent and severe complication is diabetic neuropathy, which causes ulcers, gangrene, and amputations, especially in the presence of an infection [17,18,19,20,21,22,23,24,25,26,27].

By increasing glycosylation end products, reducing cerebral blood flow, and neurotransmitter dysfunction, hyperglycemia influences cognitive function with major cardiovascular events. Psychiatric disorders, especially anxiety and depression, are more common in patients with DM, with a reduction in the ability of these patients to manage their disease and a lower QoL [23,24,25,26].

The QoL of DM patients is lower compared to the general population due to the poor control of diabetes, its complications, the fear of something unknown, the fear of family and social consequences, and the fear of going through the diagnostic stages and trying new treatments [27]. QoL is a multidimensional concept that incorporates the subjective perception of an individual’s physical, emotional, and social well-being, including both a cognitive component (satisfaction) and an emotional component (happiness). The assessment of disabilities is an indicator of the general health status, and their measurement is directly influenced by the number and severity of chronic diseases present, central elements of the QoL [28,29,30,31].

The main objectives of T2DM management are preventing acute and chronic complications, improving the QoL, and increasing life expectancy. Thus, the main purpose of this study was to evaluate the QoL of patients with T2DM, identify the factors with the greatest impact, and identify the interventions required to improve it.

## 2. Materials and Methods

### 2.1. Study Design and Patients

Patients previously diagnosed with T2DM, routinely monitored at 3 or 6 months through the Diabetes Center of the “Pius Brinzeu” County Emergency Hospital Timisoara, were prospectively enrolled in a cross-sectional study between January and May 2024. The inclusion criteria were diagnosis of T2DM, Hb > 7 g/dL, BMI ≥ 18 kg/m^2^, and age > 18 years. The exclusion criteria were other forms of diabetes, age < 18 years, BMI < 18 kg/m^2^, a severe form of anemia (Hb ≤ 7 g/dL), severe liver and kidney damage, neurological conditions, or other psychiatric conditions. Patients were of average economic status, either employed or retired. The research was conducted in accordance with the Declaration of Helsinki (2013 version). All patients provided informed consent. The Ethics Committee of Timisoara County Emergency Hospital approved the study protocol (416/15 November 2023).

### 2.2. Clinical and Paraclinical Assessments

In the study, we assessed age; gender; duration of T2DM; and anthropometric indexes such as weight, height, and body mass index (BMI). In addition, other biological parameters, including the lipid panel, were assessed. The serum creatinine, estimated glomerular filtration rate (eGFR), and urine albumin/creatinine ratio (UACr) were measured to assess renal function. Fasting glycemia (FG), postprandial glycemia (PPG), and glycated hemoglobin (HbA1c) were utilized to evaluate glycemic control.

We screened for sensitive symmetric, distal diabetic polyneuropathy (DPN) by studying the nerve conduction velocity (NCV) at the common peroneal nerve, a sciatic nerve branch responsible for movement and sensation in the lower leg, foot, and toes. To determine the peroneal nerve’s NCV, we used the portable electromyograph Neuro-MEP-Micro—Neurosoft France (version 2009). We studied the sensory and motor nerve conduction, F-wave, H-reflex, and motor and sensory investigation through electroneuromyography. Dysfunction of the peroneal nerve causes peripheral neuropathy lesions and is diagnosed if NCV < 40 m/s [32]. Also, we assessed the presence of heart failure (HF) and cerebral ischemic stroke from the patient’s history and medical documents. Antihyperglycemic treatment was also recorded as non-insulinic therapies: metformin, sulfonylureas, sodium-glucose cotransporter-2 inhibitors (ISGLT2), incretins: GLP-1-receptor agonists (AR-GLP1), dipeptidyl peptidase-4 inhibitors (IDPP4), and insulin therapies in various combinations.

We assessed the QoL using the EQ-5D-3L questionnaire, introduced in 1990 by an international group of researchers—EuroQol Group. The EQ-5D-3L contains a descriptive self-rating system that quantifies health status on a scale from 0 (deceased) to 1 (in perfect health), taking into account the individual’s perception of 5 dimensions: mobility, self-care capacity, the ability to carry out daily activities, the existence of pain or discomfort, and anxiety or depression. Each dimension includes three levels: no difficulty (level 1), some difficulty (level 2), and extreme difficulty (level 3). The tool additionally has a comprehensive health scale, wherein the rater assigns a numerical value ranging from 1 to 100 to characterize their health status, with 100 representing the highest conceivable state [33].

### 2.3. Statistical Analysis

The statistical analysis was conducted using MedCalc^®^ Statistical Software version 22.016, developed by MedCalc Software Ltd. in Ostend, Belgium, and Microsoft Office Excel Professional Plus 2021 version 2406. The continuous variables were tested for normal distribution with the Shapiro–Wilk test and described according to their distribution using the mean with standard deviation for normally distributed variables and the median with rank, or 25–75 percentiles values, for the non-parametric variables. Both percentages and absolute values were displayed for the category variables. The comparison between two sets of continuous, non-normally distributed variables was done using the Mann–Whitney test. A correlation study and multivariate regression analysis (using the enter method and then the stepwise method to check and possibly remove variables that became non-significant) were carried out to ascertain the direction and degree of the relationships between the numerical variables. The minimum required sample size for a correlation coefficient of 0.2 with the probability of making a type I error (α-level 0.05, two-sided) and a probability of making a type II error (β-level 0.10) was 258 subjects. Odds ratio (OR) analysis was used to measure the strength of an association between two events (the presence of HF, DPN, stroke, and insulin therapy) and a reduced EQ-5D-3L score < 50.

We provided the 95% confidence interval for the statistical analyses and deemed a *p*-value below 0.05 statistically significant.

## 3. Results

Following the inclusion criteria, this study included 299 T2DM patients with a median age of 62.88 (57; 70) years and median diabetes duration of 10 (6; 15) years, of which 37.1% were men (111/299) and 62.9% were women (188/299). The patients exhibited suboptimal glycemic control, evidenced by a median HbA1c of 8% (7; 9.3). Men had a significantly higher weight than women, but overall, the BMI was similar in both sexes. Women had a lower median eGFR of 73 mL/min compared to men with a median eGFR of 95 mL/min (*p* < 0.0001) (Table 1).

**Figure 1:**
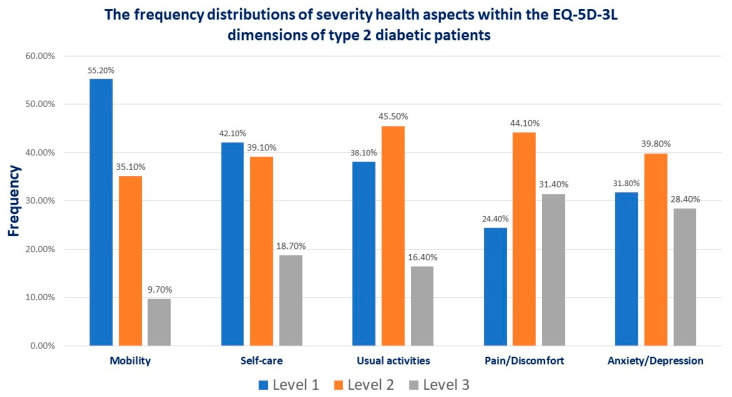
The frequency distribution of severity health aspects within the EQ-5D-3L dimensions of type 2 diabetes patients attending the Diabetes Centre from “Pius Brinzeu” County Emergency Hospital Timisoara, Romania, in 2024.

In the study group, 9.7% presented extreme difficulty (level 3 EQ-5D-3L) in mobility, 18.5% declared severe difficulty in self-care (level 3 EQ-5D-3L), and 16.4% in usual activities (level 3 EQ-5D-3L). One-third (28.4%, respectively, or 31.4%) of the patients presented severe pain or discomfort, respectively, or severe anxiety or depression (level 3 EQ-5D-3L). In 35–45% of the cases, patients with T2DM presented level 2 health conditions within the EQ-5D-3L dimensions (Figure 1).

**Figure 2:**
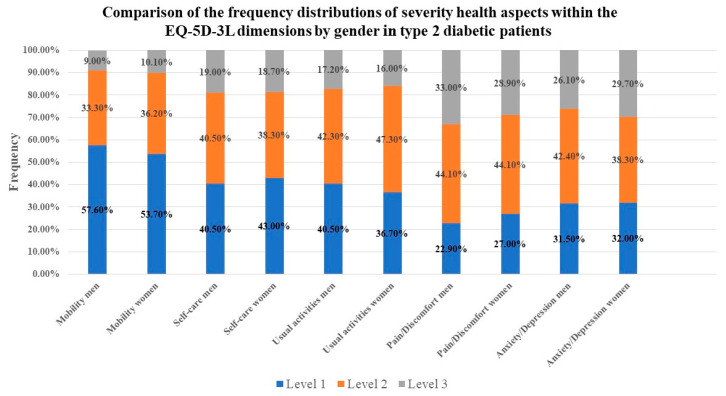
The comparison of the frequency distribution of severity health aspects within the EQ-5D-3L dimensions by gender in type 2 diabetes patients attending the Diabetes Centre from “Pius Brinzeu” County Emergency Hospital Timisoara, Romania, in 2024.

No significant differences were found between genders in the frequency distributions of severity health aspects within the EQ-5D-3L dimensions (Figure 2).

The patients were asked how they evaluated their health on a vertical visual analog scale, between 0% (worst imaginable) and 100% (best imaginable health state). The mean EQ-5D-3L score was 55%. There were no statistically significant differences among genders; in women, the score was 60% (40; 70), and in men, 50% (40; 70), *p* > 0.05.

**Figure 3:**
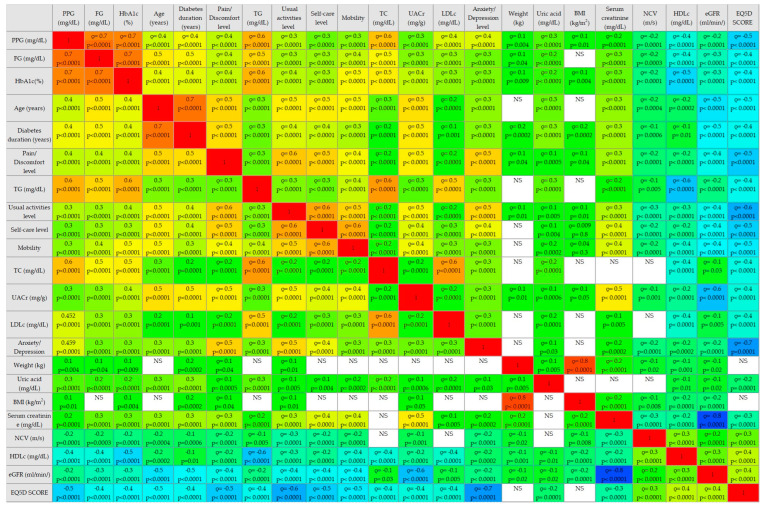
Correlogram of the EQ-5D-3L score. Abbreviations: DM, diabetes mellitus; BMI, body mass index; FG, fasting glycemia; PPG, postprandial glycemia; HbA1c, glycated hemoglobin; TG, total cholesterol; LDLc, low-density lipoprotein cholesterol; TG, triglycerides; HDLc, high-density lipoprotein cholesterol; eGFR, estimated glomerular filtration rate; UACr, urinary albumin/creatinine ratio; NCV, nerve conduction velocity; ρ = Spearman’s rank correlation coefficient. Positive correlations are displayed in blue, and negative correlations are in red. NS: statistically not significant results (*p* > 0.05); *p* < 0.05 are statistically significant. The intensity of the colors indicates the power of correlation.

The EQ-5D-3L score was associated with the main studied parameters in the correlation analysis. The results showed an inverse mild correlation with uric acid (ρ = −0.2, *p* < 0.0001) and serum creatinine (ρ = −0.3, *p* < 0.0001); an inverse moderate correlation with FP, HbA1c, TG, TC, LDLc, UACr, diabetes duration (ρ = −0.4, *p* < 0.0001), PPG, age, mobility, self-care, and pain/discomfort (ρ = −0.5, *p* < 0.0001); an inverse strong correlation with usual activities (ρ = −0.6, *p* < 0.0001) and anxiety/depression (ρ = −0.7, *p* < 0.0001); and a direct mild correlation with NCV (ρ = 0.3, *p* < 0.0001), HDLc, and eGFR (ρ = 0.4, *p* < 0.0001), as presented in the correlogram (Figure 3). These findings suggest that altered glycemic control or lipid profile, progression of renal impairment, older age, longer diabetes duration, and altered NCV are negative factors for patients’ quality of life, who more often experience altered mobility, pain or discomfort, and anxiety or depression and have challenges in quality self-care.

**Figure 4:**
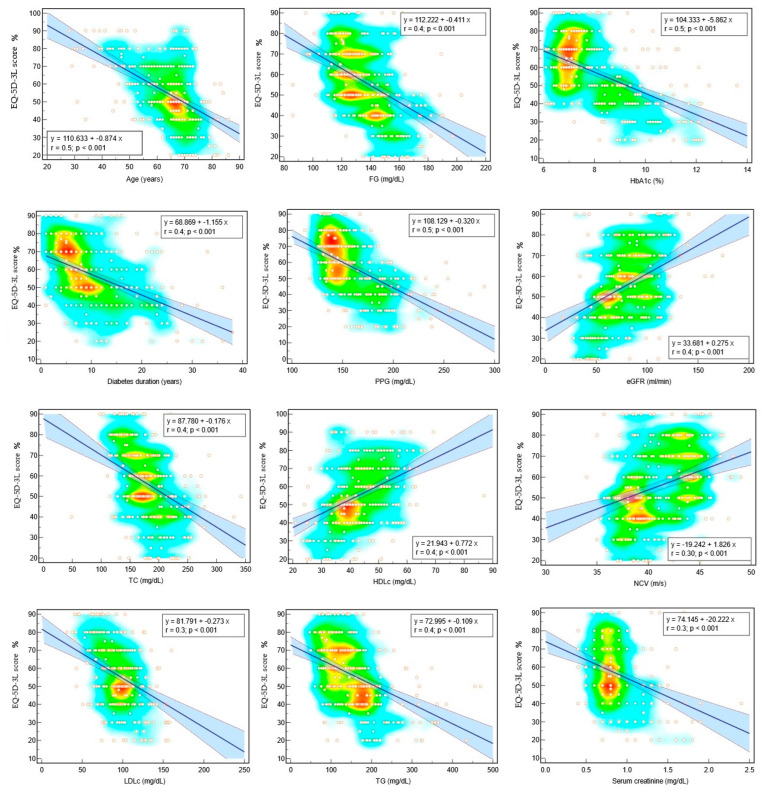
Scattered diagrams and regression lines of the associated factors with the EQ-5D-3L score.

We applied multivariate regression analysis to evaluate how a value of one parameter in a linear equation could predict the values of the EQ-5D-3L score and presented the results in Table 2 and scattered diagrams in Figure 4. A higher EQ-5D-3L score was associated with younger age (*p* = 0.002); shorter diabetes duration (*p* = 0.03); lower PPG (*p* 0.0001), TC (*p* = 0.04), and weight (*p* = 0.02); and higher HDLc (*p* = 0.01), eGFR (*p* = 0.04), and NCV (*p* = 0.01). The results indicate that younger patients recently diagnosed with diabetes with good postprandial glucose control, lower weight, and optimal values of HDLc and renal function have a better quality of life.

**Figure 5:**
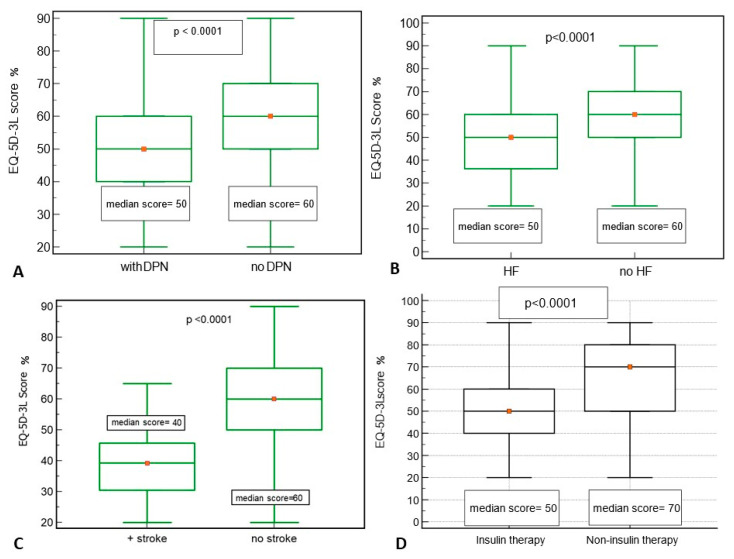
Comparison of the EQ-5D-3L score by the presence of diabetic polyneuropathy (**A**), heart failure (**B**), ischemic cerebral stroke (**C**), and insulin therapy versus non-insulin regimes (**D**) in studied patients with T2DM.

In the study group, 43.8% (131/299) patients had DPN, 42.5% (127/299) had HF, 11.03% (33/299) had a history of cerebral stroke, and 58.5% (175/299) of patients were treated with insulin. Patients with DPN, HF, or stroke had a significantly lower median EQ-5D-3L score of 50% (Figure 5A,B), respectively, or 40% (Figure 5C). The insulin group presented a median EQ-5D-3L of 50% (40; 60), significantly reduced compared to the patients treated with non-insulin regimens, who had a median EQ-5D-3L of 70% (50; 80) (Figure 5D). We performed the OR analysis to measure the strength of association between the presence of HF, DPN, stroke, and insulin therapy and the likelihood of reduced quality of life (EQ-5D-3L < 50%). All studied factors increase the likelihood of a reduced quality of life (EQ-5D-3L < 50%). The results are shown in Table 3 and Figure 5 below.

## 4. Discussion

Quality of life (QoL) has emerged as a crucial objective in disease management due to the shift in the medical approach from a biomedical model centered on the disease to a more holistic biopsychosocial model focused on patient well-being. According to recent research, complications related to diabetes, fear of hypoglycemia, and lifestyle changes are among the primary factors contributing to a decrease in QoL for patients with DM. Other factors, including advanced age, female sex, anxiety/depression, comorbidities, and insulin therapy, have also been identified as predictors of decreased QoL for patients with DM [34,35,36].

QoL is evaluated by considering the disease’s impact on daily activities, vitality, mobility, and work capacity [37]. In patients with T2DM, specific elements that are assessed to determine QoL include symptoms, fears, adherence to treatment and diet, and social and family support [38,39]. The QoL assessment did not include sociodemographic factors (marital status, gender, education, area of residence, monthly income, and religiosity). We followed the impact of the disease with its complications on the patient’s ability to take care of themselves without symptoms and without developing depression/anxiety.

There is an inverse relationship between the number of DM complications and QoL [40,41]. Donald et al. reported poor QoL in diabetic patients with multiple complications, especially if they were also associated with psychiatric disorders, such as anxiety or depression [42].

The EQ-5D-3L questionnaire is a tool based on preferences that assess health based on indices and values. The three-level versions are level 1: “no problems”, level 2:, “some problems”, and level 3: “severe problems”. In our study, almost half of the patients with T2DM had some problems, which places them in level 2 of the EQ-5D-3L. Approximately 10% of the patients presented extreme mobility difficulties (level 3), and 20% had severe self-care difficulties.

The questionnaire also includes an analog scale of self-perception of health, scored from 0 to 100: 0 representing the worst state and 100 the best state of health. In our group, the average score was 55%, with no significant difference between the two sexes: for women, the score was 60%, and for men, 50%; *p* > 0.05.

Overweight or obese patients with a higher BMI have a poorer QoL. Obesity has become one of the greatest challenges of our century, a burden for individuals and health systems worldwide [43]. A meta-analysis that included 18 studies and over 50,000 patients with T2DM showed that a sedentary lifestyle, increased consumption of red meat, a long duration of DM, the presence of hypertension, and suboptimal glycemic control are associated with a poor quality of life. Moreover, additional variables, including smoking habits, obesity, and elevated cholesterol levels, have been shown to have detrimental effects on the quality of life experienced by individuals with T2DM. Implementing healthy lifestyle adjustments, such as engaging in regular physical activity, maintaining a well-balanced diet, and employing stress management strategies, may enhance the quality of life for those diagnosed with T2DM [44].

Usually, patients with DM have numerous associated comorbidities. A study from the Netherlands by Wermeling et al. that included 2086 T2DM patients demonstrated that those with comorbidities have a lower QoL than those without comorbidities [45]. Young people know how to enjoy life and are healthier than older people, so there is an inverse relationship between age and QoL. Studies conducted in different countries have shown that older T2DM patients have a lower QoL. Prolonged and complicated treatments lead to adverse effects and poor QoL among elderly patients [46,47,48]. Furthermore, in our study, patients with chronic kidney disease, ischemic coronary disease, stroke, and polyneuropathy had a poorer QoL.

Chronic DM complications were present in a large number of patients included in the study: 43.8% had DN, 42.5% HF, and 11.03% stroke. Those with complications had a significantly lower score regarding self-assessment of health. We also assessed QoL depending on the antidiabetic treatment. Thus, patients treated with insulin had a significantly lower score than those treated with non-insulin medication: 50% compared to 70% (*p* < 0.05).

HbA1c serves as an indicator of glycemic control and the potential for complications in patients with DM. In a study carried out at the Diabetes Center of Melk Hospital in Austria by Nawras Al-Taie et al., it was demonstrated that there exists a significant and inverse relationship between the HbA1c value and QoL in individuals with DM [49]. Similarly, in our study, poor glycemic control, dyslipidemia, impaired renal function, elevated uric acid levels, and the presence of chronic complications all had a detrimental impact on QoL, manifesting as difficulties in mobility, self-care, pain, anxiety, or depression.

There are primarily five categories of factors that are essential for maintaining good health and a high quality of life. These include (1) lifestyle; (2) healthy eating habits (encompassing diet, emotional, and mental nourishment); (3) level of education; (4) self-accountability; and (5) environmental factors such as toxins, allergies, viruses, and bacteria. Among these, lifestyle, diet quality, and healthy eating habits are modifiable risk factors for physical and mental health. The Mediterranean diet, for instance, has consistently been ranked as the healthiest diet globally, owing to its ability to reduce the risk of chronic diseases and improve the overall quality of life [50,51]. Patients with diabetes, in particular, are at a heightened risk of contracting infections and experiencing a poorer prognosis. The COVID-19 pandemic posed one of the greatest challenges for these individuals, given the increased likelihood of severe complications, widespread fear, and lockdown measures [52,53,54].

The current recommendations for the treatment of T2DM advocate for the use of drugs with cardiorenal protective effects in addition to their antihyperglycemic properties. Recent clinical trial data have demonstrated cardiorenal protection for new classes of antidiabetic medications, making them a suitable option for patients with cardiovascular risk or kidney disease. The recommended treatment options are GLP-1 agonists or SGLT2 inhibitors, and other classes of medications may be used to intensify the treatment [55]. GLP-1 agonists have been shown to reduce body weight, blood sugar, blood pressure, and inflammation and improve lipid metabolism. They also reduce albuminuria and slow the progression to end stage renal disease by decreasing GFR or causing kidney destruction [56].

By simplifying treatment with oral antidiabetic drugs that have complex pathogenetic mechanisms and offer cardiovascular and renal benefits, administered once a week, patients can experience fewer adverse effects, reduce the stress generated by the disease, and improve their quality of life.

Social support at all levels of care for DM patients, promoting self-care strategies, counseling, and permanent education, and promoting a healthy lifestyle will improve the psychosocial status, treatment adherence, and QoL of T2DM patients.

### Study Limitations

The study’s limitations were that we did not compare with other patients without DM and did not evaluate the QoL in all aspects (emotions, feelings, well-being, living conditions, and income).

## 5. Conclusions

Patients’ QoL is essential, because it determines the ability to manage their disease. Patients who are aware of their diseases will have better outcomes and QoL. Many factors are involved in the QoL, but the most important are diabetes duration, chronic hyperglycemia, altered lipid profile, progression of renal impairment, and altered NCV. These represent negative factors for patients’ quality of life, who more often experience altered mobility, pain or discomfort, or anxiety or depression and have challenges in quality self-care. Moreover, stroke, HF, and neuropathic complications have a negative impact on QoL.

## Figures and Tables

**Table 1:** The general characteristics of the patients, compared by gender.

Variable	Overall	Men (*n* = 111)	Women (*n* = 188)	
	Median (25–75 Percentiles)	Median (Average Rank)	Median (Average Rank)	*p* ^a^
Age (years)	66 (57; 70)	65 (142.5)	66 (149.6)	0.4
DM duration (years)	10 (6; 15)	10 (144.7)	10 (148.3)	0.7
Weight (kg)	82 (72; 93)	89 (184.9)	79 (124.5)	<0.0001
BMI (kg/m^2^)	29.1 (26; 33)	30 (149.3)	29 (145.6)	0.7
FG (mg/dL)	135 (122; 150)	137 (148.3)	135 (146.2)	0.8
PPG (mg/dL)	156 (140; 184.7)	153 (145.5)	158 (147.8)	0.8
HbA1c (%)	8 (7; 9.3)	7.9 (142.4)	8.1 (149.7)	0.4
TC (mg/dL)	180 (152; 205)	182 (155.9)	178 (141.7)	0.1
LDLc (mg/dL)	96 (77; 110)	97 (153.8)	95 (142.9)	0.2
TG (mg/dL)	147 (104; 187.7)	143 (147.5)	151.5 (146.6)	0.9
HDLc (mg/dL)	43 (37; 51)	42.8 (150.6)	43 (144.8)	0.5
Serum creatinine (mg/dL)	0.8 (0.7; 1)	0.9 (160.5)	0.8 (138.9)	0.03
Uric acid (mg/dL)	4.7 (3.7; 5.9)	4.9 (160.7)	4.6 (138.8)	0.03
UACr (mg/g)	32 (12; 81.2)	31 (141.9)	36.2 (150)	0.4
eGFR (mL/min)	82 (60; 100)	95 (175.3)	73 (130.2)	<0.0001
NCV (m/s)	40.6 (38.6; 43.7)	40.3 (141)	41 (149.7)	0.3

Abbreviations: DM, diabetes mellitus; BMI, body mass index; FG, fasting glycemia; PPG, postprandial glycemia; HbA1c, glycated hemoglobin; TG, total cholesterol; LDLc, low-density lipoprotein cholesterol; TG, triglycerides; HDLc, high-density lipoprotein cholesterol; eGFR, estimated glomerular filtration rate; UACr, urinary albumin/creatinine ratio; NCV, nerve conduction velocity. ^a^ Mann–Whitney test for sex differences, *p* < 0.05 statistically significant. Continuous variables (with non-Gaussian distribution) are described by their median (interquartile range in men and women, or 25–75 percentiles overall).

**Table 2:** Multivariate regression analysis models of the EQ-5D-3L score and associated factors.

Independent Variables	Coefficient	Std. Error	95% CI	t	*p*	r_partial_	r_semipartial_
Enter method, R^2^-adjusted = 0.48, Multiple correlation coefficient = 0.71, *p* < 0.0001							
(Constant)	57.5411	21.5172	15.1871 to 99.8951	2.6742	0.0079		
Age	−0.3565	0.1147	−0.5823 to −0.1307	−3.1074	0.0021	−0.1816	0.1292
Diabetes_duration	−0.3425	0.1643	−0.6660 to −0.01898	−2.0838	0.0381	−0.1229	0.08663
HbA1c	−0.7113	0.9000	−2.4829 to 1.0602	−0.7904	0.4300	−0.04693	0.03286
FG	0.1621	0.06878	0.02668 to 0.2974	2.3563	0.0191	0.1387	0.09796
PPG	−0.1871	0.04648	−0.2785 to −0.09557	−4.0247	0.0001	−0.2327	0.1673
BMI	0.6662	0.3947	−0.1107 to 1.4432	1.6878	0.0925	0.09983	0.07017
HDLc	0.2546	0.1084	0.04119 to 0.4680	2.3484	0.0195	0.1383	0.09763
TC	−0.03567	0.02984	−0.09441 to 0.02307	−1.1953	0.2330	−0.07088	0.04969
LDLc	−0.07466	0.04037	−0.1541 to 0.004807	−1.8493	0.0655	−0.1093	0.07688
Serum_creatinine	−6.7512	5.0417	−16.6752 to 3.1728	−1.3391	0.1816	−0.07935	0.05567
Uric_acid	0.07755	0.2652	−0.4445 to 0.5996	0.2924	0.7702	0.01738	0.01216
Weight	−0.07698	0.1210	−0.3151 to 0.1612	−0.6363	0.5251	−0.03780	0.02645
UACr	0.01088	0.007235	−0.003358 to 0.02512	1.5042	0.1336	0.08906	0.06253
eGFR	0.06192	0.05964	−0.05548 to 0.1793	1.0382	0.3000	0.06160	0.04316
NCV	0.6841	0.2844	0.1242 to 1.2439	2.4049	0.0168	0.1415	0.09998
Stepwise method, R^2^-adjusted = 0.47, Multiple correlation coefficient = 0.69, *p* < 0.0001							
(Constant)	96.5316	14.3615	68.2664 to 124.7968	6.7215	<0.0001		
Age	−0.5003	0.08287	−0.6634 to −0.3372	−6.0367	<0.0001	−0.3331	0.2539
PPG	−0.1621	0.03045	−0.2220 to −0.1022	−5.3233	<0.0001	−0.2974	0.2239
HDLc	0.2040	0.09819	0.01070 to 0.3972	2.0771	0.0387	0.1207	0.08736
LDLc	−0.1209	0.03351	−0.1869 to −0.05496	−3.6081	0.0004	−0.2066	0.1518
Serum_creatinine	−7.2795	2.8203	−12.8302 to −1.7288	−2.5811	0.0103	−0.1494	0.1086
NCV	0.6461	0.2827	0.08975 to 1.2024	2.2856	0.0230	0.1326	0.09613

Abbreviations: DM, diabetes mellitus; BMI, body mass index; FG, fasting glycemia; PPG, postprandial glycemia; HbA1c, glycated hemoglobin; TG, total cholesterol; LDLc, low-density lipoprotein cholesterol; TG, triglycerides; HDLc, high-density lipoprotein cholesterol; eGFR, estimated glomerular filtration rate; UACr, urinary albumin/creatinine ratio; NCV, nerve conduction velocity; CI, confidence interval; *p* < 0.05 statistically significant.

**Table 3:** Risk analysis of the associated factors with the reduced EQ-5D-3L score.

Variable	z Statistic	Odds Ratio	95% CI	*p*
DPN	2.89	2.71	1.60–4.59	0.0002
HF	5.37	2.79	1.64–4.72	0.0001
Cerebral stroke	4.71	7.05	3.12–15.88	<0.001
Insulin therapy	4.12	3.70	2.00–7.04	<0.001

Abbreviations: DPN—diabetic polyneuropathy; HF—heart failure; the reduced EQ-5D-3L score is considered below 50% in the present study.

## Data Availability

Patients who participated in this study did not provide written consent for the public dissemination of their data; therefore, supporting data are unavailable due to the sensitive nature of the research.
